# Patient-Reported Experiences of Breast Cancer Screening, Diagnosis, and Treatment Delay, and Telemedicine Adoption during COVID-19

**DOI:** 10.3390/curroncol29080467

**Published:** 2022-08-20

**Authors:** Simo Du, Laura Carfang, Emily Restrepo, Christine Benjamin, Mara M. Epstein, Ricki Fairley, Laura Roudebush, Crystal Hertz, Leah Eshraghi, Erica T. Warner

**Affiliations:** 1 SurvivingBreastCancer.org, Boston, MA 02119, USA; 2 Mongan Institute, Clinical Translational Epidemiology Unit, Massachusetts General Hospital, Boston, MA 02114, USA; 3 SHARE Cancer Support, New York, NY 10036, USA; 4 Meyers Health Care Institute, a Joint Endeavor of the University of Massachusetts Medical School, Fallon Health, and Reliant Medical Group, Worcester, MA 01605, USA; 5 Division of Geriatric Medicine, Department of Medicine, University of Massachusetts Medical School, Worcester, MA 01655, USA; 6 TOUCH, The Black Brest Cancer Alliance, Annapolis, MD 21403, USA; 7 Dr. Susan Love Foundation for Breast Cancer Research, West Hollywood, CA 90069, USA

**Keywords:** breast cancer, COVID-19, mammography, health disparities

## Abstract

Purpose: To evaluate and quantify potential sociodemographic disparities in breast cancer screening, diagnosis, and treatment due to the COVID-19 pandemic, and the use of telemedicine. Methods: We fielded a 52-item web-based questionnaire from 14 May 2020 to 1 July 2020 in partnership with several U.S.-based breast cancer advocacy groups. Individuals aged 18 or older were eligible for this study if they: (1) received routine breast cancer screening; OR (2) were undergoing diagnostic evaluation for breast cancer; OR (3) had ever been diagnosed with breast cancer. We used descriptive statistics to understand the extent of cancer care delay and telemedicine adoption and used multivariable logistic regression models to estimate the association of sociodemographic factors with odds of COVID-19-related delays in care and telemedicine use. Results: Of 554 eligible survey participants, 493 provided complete data on demographic and socioeconomic factors and were included in the analysis. Approximately half (n = 248, 50.3%) had a personal history of breast cancer. Overall, 188 (38.1%) participants had experienced any COVID-19-related delay in care including screening, diagnosis, or treatment, and 339 (68.8) reported having at least one virtual appointment during the study period. Compared to other insurance types, participants with Medicaid insurance were 2.58 times more likely to report a COVID-19-related delay in care (OR 2.58, 95% Cl: 1.05, 6.32; *p* = 0.039). Compared to participants with a household income of less than USD 50,000, those with a household income of USD 150,000 or more were 2.38 (OR 2.38, 95% Cl: 1.09, 5.17; *p* = 0.029) times more likely to adopt virtual appointments. Self-insured participants were 70% less likely to use virtual appointment compared to those in other insurance categories (OR 0.28, 95% Cl: 0.11, 0.73; *p* = 0.009). Conclusions: The COVID-19 pandemic has had a significant impact on breast cancer screening, diagnosis, and treatment, and accelerated the delivery of virtual care. Lower-income groups and patients with certain insurance categories such as Medicaid or self-insured could be more likely to experience care delay or less likely to use telemedicine. Careful attention must be paid to vulnerable groups to insure equity in breast cancer-related service utilization and telemedicine access during and beyond the COVID-19 pandemic.

## 1. Introduction

The novel severe acute respiratory syndrome coronavirus 2 (SARS-CoV-2), which causes the disease coronavirus 2019 (COVID-19), led to a global pandemic that in addition to the high toll of mortality and morbidity from the disease, has disrupted healthcare systems [1] and cancer care around the world [2,3]. Beginning in March 2020, U.S. federal, state, and local government regulations led to mammography clinic closures or operation at diminished capacity to preserve personal protective equipment, prevent viral transmission in health care facilities, and allow for clinical staff redeployment [1,4,5]. Database analyses show that this led to a sharp decline in routine breast cancer screening in Spring 2020, with data from Epic medical records systems showing a 94% decline in March 2020 [6,7]. However, while declines in screening have been documented, relatively little data has been collected from the patient perspective to understand their experience of pandemic-related delays and how it may differ for different sociodemographic groups. Individuals of lower socioeconomic status and communities of color have been disproportionately burdened by the health, social, and economic consequences of the COVID-19 pandemic [8]. Rural communities have less reserve capacity to respond to the ongoing COVID-19 crisis while also addressing non-COVID-19 health needs [9]. For these traditionally medically underserved populations with already limited access to preventive health services, existing disparities may only be exacerbated by COVID-19 related disruptions.

Beyond screening and diagnosis, the pandemic has also affected care for individuals with breast cancer. Current research suggests cancer patients are more likely to experience worse outcomes due to COVID-19 [10,11,12,13]. As a result, breast cancer patients and their providers have had to carefully weigh the risks of contracting COVID-19 with the benefits of receiving routine surveillance tests and life-prolonging cancer therapies [14]. The pandemic has necessitated changes to how oncologists approach cancer care and cancer management [15,16]. These changes have included alterations to treatment sequence, including delaying surgery [17]; use of neoadjuvant therapy [3,18]; or omitting, delaying, or shortening the treatment plans in an effort to reduce the number of in-person visits [19]. de Joode et al. discovered that 30% of patients experienced a disruption to oncological treatment or follow up, which was mainly associated with chemotherapy and immunotherapy [14]. In accordance with the findings reported by Joode et al., Papautsky and Hamlish’s results showed 44% of participants reported cancer care delays or interruptions due to the COVID-19 pandemic [20]. Healthcare providers, alongside their patients, have reassessed the delivery of cancer treatment and follow-up care [16] through innovative techniques to reduce COVID-19 transmission without compromising treatments that could deteriorate one’s condition and increase the risk of relapse [21]. While the literature on cancer care in the time of COVID-19 continues to grow, there is a dearth of research on how the health pandemic is directly impacting those diagnosed with breast cancer.

In light of these rapid changes, healthcare providers adopted telemedicine for routine visits, follow-up appointments, and outpatient consultations rather than in-person encounters, when possible, to reduce potential exposure to COVID-19 [17,18]. In the COVID-19 era, healthcare systems have been extremely taxed and overloaded with patients that have the virus and need intensive monitoring, thus requiring healthcare administration to reallocate resources, both human as well as material [22]. It is imperative that patients, researchers, and medical professionals have a robust understanding of how the COVID-19 disease has impacted (and continues to impact) individuals eligible for routine breast cancer screening, in need of follow-up after an abnormal mammogram, or with a current breast cancer diagnosis. Therefore, the purpose of this study is to understand how the COVID-19 pandemic has disrupted breast cancer screening, diagnosis, and treatment, and accelerated the adoption of telemedicine in the healthcare setting, and to evaluate the potential disparity in cancer care delay and telemedicine adoption.

## 2. Materials and Methods

### 2.1. Survey Development and Distribution

This cross-sectional web-based survey was administered between 14 May and 1 July 2020. Data were collected via REDCap, a secure online survey software program [23]. Participants accessed and completed the survey either by emailing the research team and receiving a private survey link or by clicking a public link distributed via social media by the study team through advertisements by one of the five collaborating breast cancer advocacy organizations (Dr. Susan Love Foundation for Breast Cancer Research (West Hollywood, CA, USA), SHARE Cancer Support (New York, NY, USA), SurvivingBreastCancer.org (Boston, MA, USA), Sisters Network Inc. (Houston, TX, USA), and TOUCH, The Black Breast Cancer Alliance (Annapolis, MD, USA)), or through invitations sent to women age 40–74 using ResearchMatch. (accessed on 1 August 2022.) Individuals aged 18 or older were eligible for the survey if they were: (1) receiving—or planning to receive—routine screening mammograms; (2) undergoing diagnostic evaluation for breast cancer; or (3) had ever been diagnosed with breast cancer. At the end of the survey, participants could indicate if they were willing to be recontacted by the study team and provide their name and contact information for future follow-up. This study was deemed exempt by the Mass General Brigham Human Research Committee.

The online survey was developed using questions from existing surveys [24,25] and the ASCO COVID-19 guidelines [26]. The survey asked participants questions about their experiences with breast cancer screening, diagnosis, and treatment during the COVID-19 pandemic. The survey took 10–15-min to complete on average. The survey collected information on respondent demographics; the extent to which breast cancer screening, diagnosis, or treatment had been changed or delayed because of COVID-19; personal protective practices; extent of worry about financial and health implications of COVID-19; and use of telemedicine (survey available in File S1).

### 2.2. Study Measures

#### 2.2.1. Personal History of Breast Cancer

Participants were asked: “Have you ever been told by a doctor or other healthcare provider that you had breast cancer?”. Anyone that responded ‘yes’ was considered to have a personal history of breast cancer.

#### 2.2.2. Sociodemographic Characteristics

Participants self-reported their age, race and ethnicity, U.S. region of residence, level of residential urbanicity, healthcare site, educational attainment, and household income. Race and ethnicity responses were grouped in the following categories: White, Hispanic/Latinx, Black or African American, Asian, or more than one race. Participants were asked whether they lived within one of nine U.S. regions (New England, Mid Atlantic, East North Central, West North Central, South Atlantic, East South Central, West South Central, Mountain, Pacific) or outside of the U.S. Responses were then grouped into the following categories for analysis: Northeast, Midwest, South, or West. Residential urbanicity was defined as rural, suburban, or urban. Healthcare site was defined as academic medical center, regional medical center, community hospital, or private practice. Participants were asked to report their highest degree or level of school completed and responses were dichotomized into a binary variable representing a college degree or higher (yes or no). As participants could report multiple types of health insurance, we evaluated multiple yes/no indicators of the most commonly reported insurance types including private insurance, self-insured, Medicare, Medicaid, or other insurance. Annual household income was defined as < USD 50,000, USD 50,000–74,999, USD 75,000–99,999, USD 100,000–149,999, or ≥ USD 150,000.

#### 2.2.3. Delays in Care

We evaluated the prevalence and predictors of self-reported delays in care including breast cancer screening, diagnosis, or treatment delay. Participants were first asked: “Have any of your doctor’s visits or treatments been changed, delayed, rescheduled, interrupted, or stopped as a result of COVID-19?”. Anyone that responded ‘yes’ to that question was considered to have experienced a COVID-19 related change in care and was then asked: “What types of changes, delays, or rescheduling have occurred as result of COVID-19, if any?” Participants that indicated that their breast cancer screening, diagnosis, or treatment had been: (1) delayed by less than 2 weeks; (2) delayed by more than 2 weeks; (3) delayed and participant did not know when it would be rescheduled; (4) or cancelled without expectation of rescheduling, were considered to have experienced a COVID-19-related care delay.

Participants were then asked to identify what specific type of care had been delayed or cancelled. Options included: mammogram, MRI, ultrasound, surgery, radiation, chemotherapy, pills/hormone therapy, targeted therapy or infusion but not chemotherapy, or clinical trial. Responses of mammogram, MRI, or ultrasound were categorized as screening delays. Responses of surgery, radiation, chemotherapy, pills/hormone therapy, targeted therapy or infusion but not chemotherapy, or clinical trial were categorized as treatment delays.

#### 2.2.4. Telemedicine Use

Participants were asked two questions: “Have your doctors added additional services to reduce in person appointments?” and “Have you or your doctors used any of the following methods to connect during the COVID-19 pandemic?”. Response options included: virtual appointments (video conferences), email exchanges, sending pills to you through the mail, or no extra services. Telemedicine use was defined as use of ‘virtual appointments (video conferences)’. We also evaluated use of alternative healthcare delivery methods including telephone, email, and receiving medication by mail.

### 2.3. Statistical Analysis

Patients’ social demographic characteristics and primary and secondary outcomes were summarized using descriptive statistics including frequency and proportion and stratified by patients’ cancer status. To estimate the association between social-demographic factors with any cancer care delay and telemedicine use, two separate multivariable logistic regression models were constructed. Covariates included breast cancer diagnosis, age, ethnicity, U.S. region, level of urbanization, healthcare site, college degree, house income, and insurance status to adjust for different patients’ characteristics. We calculated odds ratios (ORs) and 95% confidence intervals (CIs) using maximum likelihood estimation. We hypothesized that there are statistically significant associations between certain patients’ sociodemographic characteristics and cancer care delay or telemedicine use. To address the issue of missing data, a missing indicator approach was utilized and adjusted for the missing category in the multivariable logistic regression models. All analyses were conducted using STATA (Version 15; StataCorp LLC, College Station, TX, USA) and SAS software (version 9.4; SAS Institute, Cary, NC, USA). All tests were two-sided and an alpha level of 0.05 was used.

## 3. Results

### 3.1. Study Population

**Figure 1:**
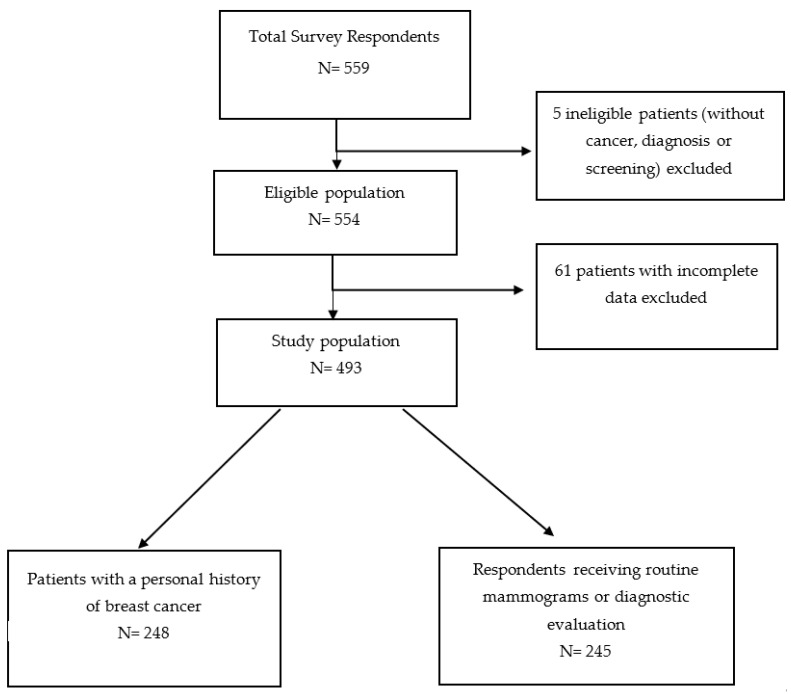
Population flowchart.

There were 559 survey respondents, 554 of whom were eligible for our survey (Figure 1). Of 554 eligible survey participants, 493 provided complete data on demographic and socioeconomic factors and were included in the analysis. Approximately half (n = 248, 50.3%) were diagnosed with breast cancer and 245 (49.7%) received or plan to receive routine screening mammograms or diagnostic evaluation for breast cancer. The demographic and socioeconomic characteristics of the population overall and by cancer status are summarized in Table 1. Most participants were between 40 to 70 years old (74.8%), White (56.6%) or Black (23.3%) race, lived in an urban (40.0%) or suburban area (47.9%), had a college degree (74.2%), and had private health insurance (60.9%). Participants with a personal history of breast cancer were older, more likely to be white, and have a college degree compared to participants without a personal history of cancer.

### 3.2. Impact of COVID-19 on Cancer Screening, Care, and Telemedicine Use

**Figure 2:**
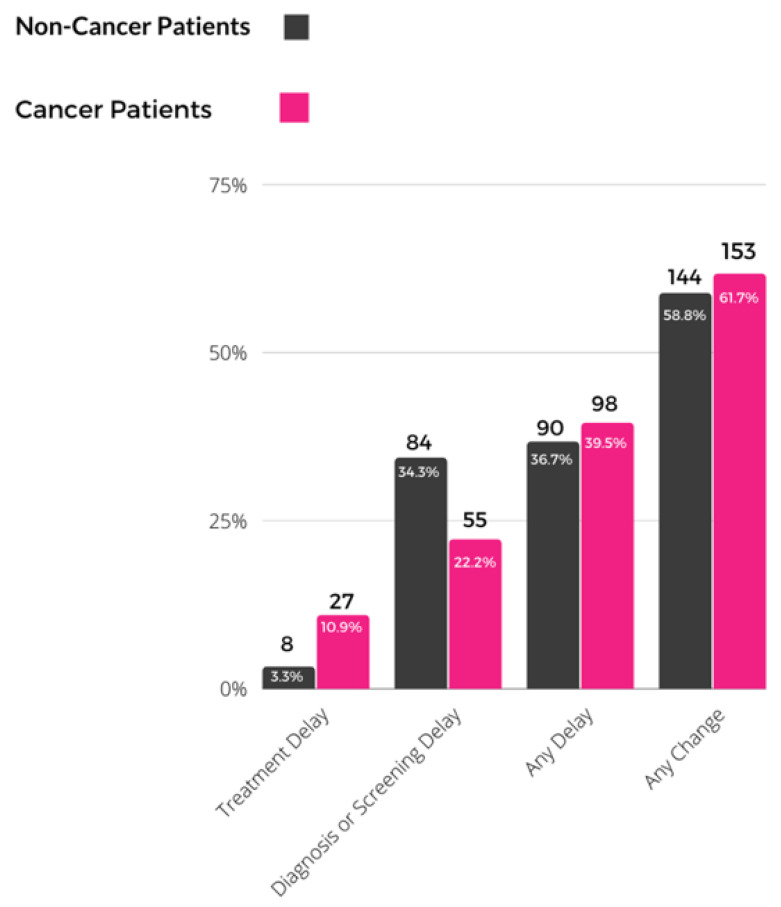
Cancer screening, diagnosis and treatment delay among survey participants.

Of the 493 participants included in the analysis, around 60% (61.7% among the cancer patients and 58.8% among the non-cancer participants) reported having any COVID-19 related change in care. Ninety (36.7%) non-cancer patients and 98 (39.5%) cancer patients reported a COVID-19-related delay in care. Among 248 cancer patients, 27(10.9%) patients reported treatment delay, while among 245 non-cancer patients, 55 (22.2%) reported any diagnosis or screening delay (Figure 2).

**Figure 3:**
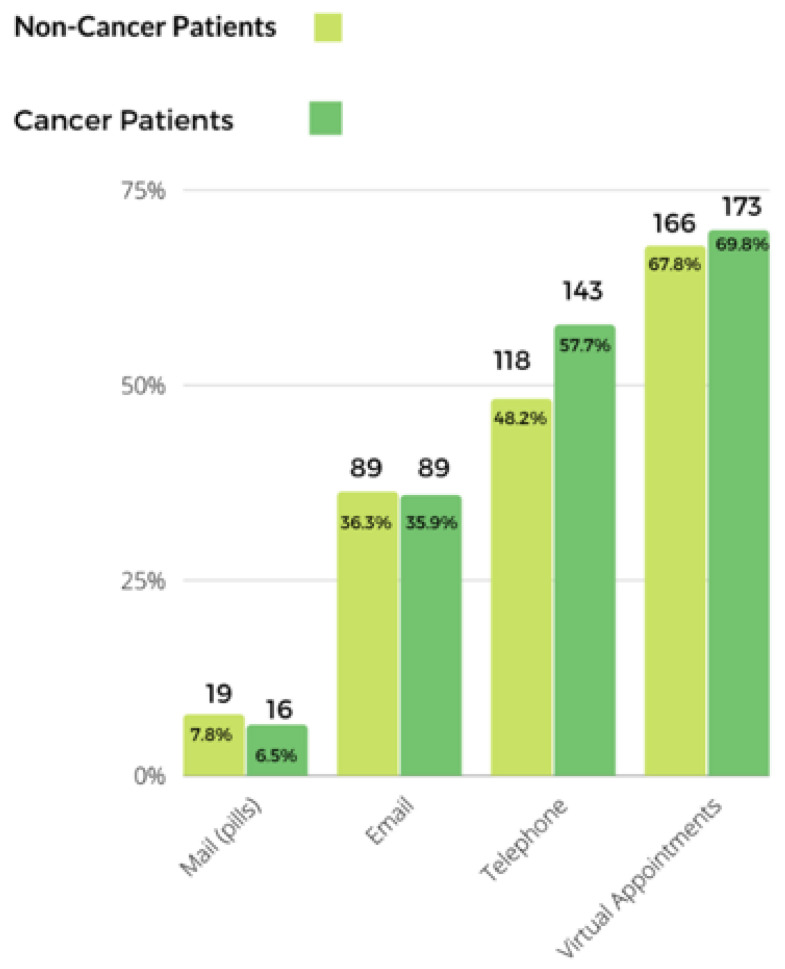
Telemedicine use and non-contact medication delivery among survey participants.

Regarding telemedicine use, over two-thirds of the participants (69.8% non-cancer and 67.8% cancer patients) reported virtual appointment utilization, while approximately half of the participants reported telephone use (48.2% non-cancer and 57.7% cancer patients) to communicate with their providers during the pandemic period. Email communication rate was relatively lower with around 36.1% reporting email use to communicate with their providers. Finally, there were 35 (7.1%) patients who reported receiving their prescribed medication through the mail (Figure 3).

### 3.3. Factors Associated with COVID-19-Related Delays in Care and Telemedicine Use

Predictors of COVID-19-related delays in care are summarized in Table 2. Odds of any COVID-19-related delay in care did not differ by age, race/ethnicity, education, household income, region, or location of care. Participants with Medicaid insurance had more than two times greater odds of experiencing delayed care compared to those who did not have Medicaid insurance (OR: 2.58, 95% CI 1.05, 6.32, *p* = 0.039).

We did not find any significant differences in telemedicine use according to age group, race/ethnicity, geographic region, areas, location of care, or education (Table 3). However, patients who reported higher annual household income had higher odds of telemedicine use compared to those with lower income. Individuals with a reported annual household income of USD 100,000–149,999 were 2.15 (95% CI 1.01, 4.55, *p* = 0.047) times more likely to have telemedicine use while the those with USD 150,000+ household income were 2.38 (95% 1.09, 5.17, *p* = 0.029) times more likely to use telemedicine compared to households with an annual income of < USD 50,000 (P-trend, 0.423).

## 4. Discussion

The results of this study demonstrate that breast cancer screening and surveillance was the most commonly disrupted breast cancer-related care early in the COVID-19 pandemic. This was true among patients with and without a breast cancer diagnosis. Of the sociodemographic factors we examined, only insurance status was associated with COVID-19-related delays in care. Specifically, participants with Medicaid insurance were more likely to report delays than individuals with other types of insurance. In terms of telemedicine use, we observed a high adoption rate in populations with diverse sociodemographic factors across different regions. More importantly, we discovered that those with higher household income were more likely to use telemedicine, specifically, video appointments, compared to individuals from lower household income families. Additionally, we found that those self-insured were less likely to use telemedicine compared to other insurance types, which could be associated with telemedicine reimbursement methods and cost for different insurance categories.

These results are congruent with the findings of Joode et al. and Papautsky and Hamlish [14,20], which demonstrate self-reported treatment delay among patients with breast cancer. The finding is also consistent with London et al.’s discovery of significantly decreased cancer-related patient encounters during the pandemic period using real-world large-scale electronic medical record data [27]. This discovery of disparity in telemedicine use was consistent with Eberly et al., in which they discovered that older, female, Black, Latinx, and poorer patients had lower video-based care use [28]. Lower-income patients may have less access to computers, video devices, and the internet, which may lead to lower telemedicine uptake [29]. We did not observe inequality across different racial groups. This could be due to the limited number of respondents of color, particularly among those with a personal history of breast cancer, which leads to decreased precision and power to discover the potential disparity. Taken together, our results suggest a potential disparity of care delay among vulnerable populations. Even before the pandemic, patients with Medicaid coverage were significantly less likely to use cancer screening services and more likely to present with advanced-stage cancer at diagnosis and to have significantly worse survival [30]. COVID-19 could exaggerate the disparity and increase the gap between those with and without access to healthcare resources.

In this study, participants reported COVID-19-related delays in breast cancer screening, diagnosis, and treatment that, if continued, could lead to at aa later stage, diagnosis, disease progression, and ultimately cancer mortality that would not have happened in the absence of the COVID-19 pandemic [30,31]. While these data were collected in 2020, we know that COVID-19-related delays continue through the pandemic [32]. Prior modeling studies have estimated that each 4-week increase in diagnostic delay is associated with a 9–17% increase in cancer mortality [33]. Each 4-week delay in cancer surgery, radiotherapy, or systemic therapy leads to a 6–8% increase in mortality, but there is evidence of significant heterogeneity in this association according to patient characteristics such as age, comorbidity, and geographic region [34,35]. Longer delays across multiple needed treatments are also associated with greater mortality [36]. Moreover, as we observed greater delays among individuals with Medicaid insurance, and others have found disparities in COVID-19-related changes in cancer screening among racial/ethnic minorities [37], the impact could be unevenly distributed and be more severe in vulnerable populations including patients with lower socioeconomic status. COVID-19-related delays could further exacerbate pre-existing racial and socioeconomic disparities in breast cancer early detection, diagnosis, and treatment [38,39]. Although the transition of care delivery to telemedicine could help mitigate the detrimental effect of COVID-19 on cancer care, we must be aware that inequity in telemedicine uptake could exist. In this population, barriers to telemedicine access could impose an additional detrimental impact on their health, leading to a widened gap between populations with high versus low socioeconomic status.

The study has several strengths. First, it includes a heterogeneous population with multiple ethnicities, age groups, different socioeconomic statuses, and regions. Secondly, in contrast to other studies using aggregate level data to assess the impact of COVID-19 on cancer care delivery, we assessed patient-reported experience to reflect the direct impact on cancer care at the individual level. Moreover, we included both patients diagnosed with cancer and non-cancer patients to evaluate the impact of the pandemic on both cancer treatment and prevention. However, it is worth noting that the study has a few limitations. Firstly, the sample size of our study is small, and the respondents may not fully represent the experience of individuals with breast cancer or eligible for breast cancer screening due to information bias (i.e., participants self-reported their experience) and selection bias due to how the survey was administered (i.e., using computers, mobile phones, and technology) and who chose to participate. Similarly, in our multivariable models, the small cell counts limit the power and precision of some of our analyses. As our study is observational and we limited the number of questions to reduce participant burden, there may be residual and/or unmeasured confounding. Lastly, the cross-sectional nature of the study limits our ability to study the long-term impacts of COVID-19 and longitudinal change related to clinical outcomes. Our study occurred relatively early in the pandemic and some participants may not have yet had the opportunity to experience a delay, if for example they were not due for screening between March and July 2020. Others have been able to receive needed care after completing our survey. We did request permission to follow-up with respondents and over 75% agreed to be recontacted. We plan to survey them again to understand how their experience changed during the pandemic.

## 5. Conclusions

In conclusion, in this cross-sectional study, we discovered potential disparity regarding breast cancer-related care delays and telemedicine access for people with different household income and insurance status. Future studies are needed to confirm the finding, investigate the mechanisms, and assess the long-term outcome of breast cancer morbidity and mortality due to the delay. Moreover, as healthcare delivery shifts towards virtual care, efforts should be made to ensure the equity of telemedicine access.

## Figures and Tables

**Table 1:** Survey participant characteristics according to personal history of cancer.

Characteristic	Total N = 493	No Personal History of Breast Cancer N = 245	Personal History of Breast Cancer N = 248
**Age (years)**	**N (%)**	**N (%)**	**N (%)**
18–39	31 (6.3%)	4 (1.6%)	27 (10.9%)
40–49	104 (21.1%)	66 (26.9%)	38 (15.3%)
50–59	142 (28.8%)	79 (32.2%)	63 (25.4%)
60–69	143 (29.0%)	67 (27.3%)	76 (30.6%)
≥70	58 (11.8%)	19 (7.8%)	39 (15.7%)
Missing	15 (3.0%)	10 (4.1%)	5 (2.0%)
**Race and Ethnicity**			
White	279 (56.6%)	83 (33.9%)	196 (79.0%)
Hispanic/Latinx	28 (5.7%)	23 (9.4%)	5 (2.0%)
Black or African-American	115 (23.3%)	82 (33.5%)	33 (13.3%)
Asian	27 (5.5%)	23 (9.4%)	4 (1.6%)
More than one race	17 (3.4%)	16 (6.5%)	1 (0.4%)
Missing	27 (5.5%)	18 (7.3%)	9 (3.6%)
**Family History**			
No	333 (67.5%)	161 (65.7%)	172 (69.4%)
Yes	134 (27.2%)	70 (28.6%)	64 (25.8%)
Missing	26 (5.3%)	14 (5.7%)	12 (4.8%)
**U.S. Region**			
Northeast	146 (29.6%)	69 (28.2%)	77 (31.0%)
Midwest	67 (13.6%)	34 (13.9%)	33 (13.3%)
South	163 (33.1%)	94 (38.4%)	69 (27.8%)
West	77 (15.6%)	29 (11.8%)	48 (19.4%)
Missing	40 (8.1%)	19 (7.8%)	21 (8.5%)
**Level of Urbanicity**			
Urban	197 (40.0%)	102 (41.6%)	95 (38.3%)
Suburban	236 (47.9%)	112 (45.7%)	124 (50.0%)
Rural	46 (9.3%)	22 (9.0%)	24 (9.7%)
Missing	14 (2.8%)	9 (3.7%)	5 (2.0%)
**Healthcare Site**			
Academic center	167 (33.9%)	72 (29.4%)	95 (38.3%)
Regional center	117 (23.7%)	48 (19.6%)	69 (27.8%)
Community hospital	82 (16.6%)	52 (21.2%)	30 (12.1%)
Private practice	125 (25.4%)	73 (29.8%)	52 (21.0%)
Missing	2 (0.4%)	0 (0.0%)	2 (0.8%)
**College degree**			
No	117 (23.7%)	68 (27.8%)	49 (19.8%)
Yes	366 (74.2%)	170 (69.4%)	196 (79.0%)
Missing	10 (2.0%)	7 (2.9%)	3 (1.2%)
**Household income**			
<$50,000	107 (21.7%)	62 (25.3%)	45 (18.1%)
$50,000–$74,999	70 (14.2%)	33 (13.5%)	37 (14.9%)
$75,000–$99,999	74 (15.0%)	40 (16.3%)	34 (13.7%)
$100,000–$149,999	79 (16.0%)	37 (15.1%)	42 (16.9%)
≥$150,000	78 (15.8%)	29 (11.8%)	49 (19.8%)
Missing	85 (17.2%)	44 (18.0%)	41 (16.5%)
Insurance *			
Private insurance	300 (60.9%)	152 (62.0%)	148 (59.7%)
Self-insured	36 (7.3%)	13 (5.3%)	23 (9.3%)
Medicare	142 (28.8%)	57 (23.3%)	85 (34.3%)
Medicaid	39 (7.9%)	26 (10.6%)	13 (5.2%)
Other insurance	45 (9.1%)	24 (9.8%)	21 (8.5%)

* Patients could have multiple insurance categories and each insurance category is treated as a binary outcome.

**Table 2:** Number, prevalence and adjusted odds ratio of COVID-19-related delay in care by sociodemographic factors (n = 493).

	N of Patients with Any Care Delay	% of Patients with Any Care Delay	OR	95% CI	*p*
**Personal history of breast cancer**					
No	90	36.7%	1.00	Reference	
Yes	98	39.5%	0.99	0.63, 1.55	0.968
**Age (years)**					
18–39	13	41.9%	1.00	Reference	
40–49	38	36.5%	0.68	0.28, 1.64	0.389
50–59	60	42.3%	0.91	0.39, 2.10	0.821
60–69	49	34.3%	0.60	0.24, 1.45	0.254
≥70	21	36.2%	0.62	0.20, 1.90	0.402
P trend					0.867
**Race and Ethnicity**					
White	112	40.1%	1.00	Reference	
Hispanic/Latinx	9	32.1%	0.62	0.25, 1.56	0.311
Black or African American	42	36.5%	0.80	0.46, 1.41	0.445
Asian	6	22.2%	0.42	0.15, 1.17	0.097
More than one race	8	47.1%	1.13	0.37, 3.41	0.831
**U.S. Region**					
Northeast	55	37.7%	1.00	Reference	
Midwest	27	40.3%	1.11	0.58, 2.11	0.757
South	57	35.0%	1.15	0.68, 1.94	0.597
West	30	39.0%	1.23	0.65, 2.32	0.522
**Level of Urbanicity**					
Urban	78	39.6%	1.00	Reference	
Suburban	80	33.9%	0.74	0.48, 1.13	0.163
Rural	23	50.0%	1.29	0.64, 2.57	0.474
**Healthcare Site**					
Academic center	66	39.5%	1.00	Reference	
Regional center	53	45.3%	1.14	0.67, 1.92	0.634
Community hospital	30	36.6%	0.75	0.41, 1.36	0.344
Private practice	38	30.4%	0.65	0.38, 1.12	0.123
**College degree**					
No	49	41.9%	1.00	Reference	
Yes	134	36.6%	0.89	0.53, 1.48	0.643
**Household income**					
<$50,000	43	40.2%	1.00	Reference	
$50,000–$74,999	31	44.3%	1.27	0.64, 2.51	0.494
$75,000–$99,999	28	37.8%	0.97	0.48, 1.97	0.938
$100,000–$149,999	27	34.2%	0.84	0.40, 1.77	0.655
≥$150,000	24	30.8%	0.65	0.30, 1.39	0.268
P trend					0.562
Insurance *					
Private insurance	75	38.9%	1.00	Reference	
	113	37.7%	1.84	0.90, 3.75	0.096
Self-insured	174	38.1%	1.00	Reference	
	14	38.9%	1.94	0.76, 4.92	0.163
Medicare	135	38.5%	1.00	Reference	
	53	37.3%	1.23	0.65, 2.35	0.525
Medicaid	168	37.0%	1.00	Reference	
	20	51.3%	2.58	1.05, 6.32	0.039
Other government insurance	170	37.9%	1.00	Reference	
	18	40.0%	1.72	0.77, 3.83	0.188

* Patients could have multiple insurance categories and each insurance category is treated as a binary exposure.

**Table 3:** Number, percent and adjusted odds ratio of telemedicine use by sociodemographic factors (n = 491) ^a^.

Characteristic	N	%	OR	95% CI	*p*
**Personal history of breast cancer**					
No	166	67.7%	1.00	Reference	
Yes	173	69.8%	1.11	0.68, 1.80	0.678
**Age (years)**					
18–39	23	74.2%	1.00	Reference	
40–49	70	67.3%	0.84	0.32, 2.24	0.734
50–59	99	69.7%	0.93	0.36, 2.38	0.880
60–69	103	72.0%	1.13	0.42, 3.02	0.813
≥70	33	56.9%	0.53	0.16, 1.77	0.305
P trend					0.885
**Race and Ethnicity**					
White	192	68.8%	1.00	Reference	
Hispanic/Latinx	17	60.7%	0.72	0.29, 1.76	0.473
Black or African-American	80	69.6%	0.84	0.46, 1.51	0.553
Asian	19	70.4%	1.10	0.41, 2.92	0.855
More than one race	10	58.8%	0.68	0.22, 2.13	0.509
**U.S. Region**					
Northeast	102	69.9%	1.00	Reference	
Midwest	43	64.2%	0.94	0.48, 1.85	0.854
South	121	74.2%	1.27	0.72, 2.24	0.405
West	47	61.0%	0.74	0.38, 1.42	0.361
**Area**					
Urban	128	65.0%	1.00	Reference	
Suburban	173	73.3%	1.31	0.83, 2.05	0.247
Rural	30	65.2%	1.01	0.48, 2.09	0.988
**Healthcare Location**					
Academic center	118	70.7%	1.00	Reference	
Regional center	79	67.5%	1.06	0.60, 1.86	0.846
Community hospital	50	61.0%	0.85	0.46, 1.59	0.622
Private practice	92	73.6%	1.50	0.84, 2.68	0.169
**College degree**					
No	79	67.5%	1.00	Reference	
Yes	252	68.9%	1.02	0.99, 1.06	0.152
**Household income ^b^**					
<$50,000	62	57.9%	1.00	Reference	
$50,000–$74,999	48	68.6%	1.53	0.75, 3.11	0.241
$75,000–$99,999	51	68.9%	1.52	0.74, 3.12	0.252
$100,000–$149,999	60	75.9%	2.15 *	1.01, 4.55	0.047
≥$150,000	61	78.2%	2.38 *	1.09, 5.17	0.029
P trend					0.423
**Insurance ^c^**					
Private insurance	123	63.7%	1.00	Reference	
	216	72.0%	0.87	0.41, 1.84	0.708
Self-insured	324	70.9%	1.00	Reference	
	15	41.7%	0.28 **	0.11, 0.73	0.009
Medicare	245	69.8%	1.00	Reference	
	94	66.2%	1.12	0.56, 2.22	0.754
Medicaid	313	68.9%	1.00	Reference	
	26	66.7%	1.09	0.43, 2.79	0.853
Other government insurance	308	68.8%	1.00	Reference	
	31	68.9%	0.97	0.41, 2.26	0.938

^a^ Two people have been dropped from the model due to zero cells. ^b^ * *p* < 0.05 ** *p* < 0.01. ^c^ Patients could have multiple insurance categories and each insurance category is treated as a binary exposure.

## Data Availability

The data presented in this study are available on request from the corresponding author. The data are not publicly available to protect the privacy of participants.

## Supplementary Materials

The following are available online at https://www.mdpi.com/article/10.3390/curroncol29080467/s1, File S1: Survey.
