# Supplementary material for: Patient-Reported Experiences of Breast Cancer Screening, Diagnosis, and Treatment Delay, and Telemedicine Adoption during COVID-19
Source: Curr Oncol. 2022 Aug 20;29(8):5919–32. doi: 10.3390/curroncol29080467 (PMC9406797; doi:10.3390/curroncol29080467)
Supplement: Supplementary file 1 [file curroncol-29-00467-s001.zip › curroncol-1829440-supplementary.pdf]

Breast Cancer Diagnosis and Treatment

1. Have you ever been told by a doctor or other healthcare provider that you had breast cancer?
   - ❑ No (SKIP TO Q4)
   - ❑ Yes (CONTINUE)

2. When were you diagnosed with breast cancer? (If you are experiencing a recurrence, please choose when your breast cancer recurred.)
   - ❑ Less than one month ago
   - ❑ 1 to less than 3 months ago
   - ❑ 3 to less than 6 months ago
   - ❑ 6 months ago to less than 1 year ago
   - ❑ 1 year ago to less than 3 years ago
   - ❑ 3 years ago to less than 5 years ago
   - ❑ More than 5 years ago

3. Are you currently receiving active treatment (treatment given to cure the cancer, such as chemotherapy, radiation therapy, surgery, hormone therapy, etc.) for your breast cancer?
   - ❑ Yes (SKIP TO Q6)
   - ❑ No (SKIP TO Q9)

4. Are you currently undergoing diagnostic evaluation to determine whether you have breast cancer, such as a diagnostic mammogram, ultrasound, MRI, or biopsy, due to symptoms or an abnormal mammogram?
   - ❑ No (CONTINUE)
   - ❑ Yes (SKIP TO Q6)

5. Are you currently receiving routine screening mammograms?
   - ❑ No (INELIGIBLE)
   - ❑ Yes (SKIP to Q9)

6. Select the option that best describes your current situation:
   - ❑ I had an abnormal screening mammogram or have breast cancer symptoms (such as a lump or breast discharge) and am awaiting a diagnostic mammogram, ultrasound, or MRI **(SKIP TO Q9)**
   - ❑ I am scheduled for a diagnostic biopsy (medical test which involves removing a small amount of tissue to diagnose cancer) **(SKIP TO Q9)**
   - ❑ I have been newly diagnosed with cancer, but haven’t had surgery/therapy so far
   - ❑ I am in follow-up after surgery for Stage 1, 2, or 3 cancer
   - ❑ I am on adjuvant therapy (therapy to prevent the return of cancer, but cancer has not spread)
   - ❑ Cancer has spread and I am on systemic therapy (therapy to stop cancer progression, such as chemotherapy, radiation, hormone therapy, etc.)

7. What treatments are you currently receiving or scheduled to receive? (select all that apply)
   - ❑ My treatment plan has not been decided yet (SKIP TO Q9)
   - ❑ Surgery on part of the breast (*Lumpectomy* - An operation to remove the cancer and some normal tissue around it, but not the breast itself)
   - ❑ Surgery to remove the entire breast (*Mastectomy* - Surgery to remove all of the breast)
   - ❑ Radiation (The use of high-energy radiation from x-rays and other sources to kill cancer cells and shrink tumors)
   - ❑ Chemotherapy (Treatment that uses drugs to stop the growth of cancer cells, either by killing cells or by stopping them from dividing)
   - ❑ Pills/Hormone therapy (Treatment that adds, blocks, or removes hormones)
   - ❑ Targeted therapy/Infusion but not chemotherapy (Herceptin, Pertuzumab - drugs used to treat HER-2 positive breast cancer or immunotherapy)
   - ❑ Clinical Trial (A type of research that studies new tests and treatments and evaluates their effects on human health outcomes)

8. About how long ago did you receive your last treatment for breast cancer? (Treatment includes surgery, chemotherapy, radiation therapy, targeted therapy/infusion, or hormone therapy)
   - ❑ I have not received treatment
   - ❑ Less than one month ago
   - ❑ 1 to less than 3 months ago
   - ❑ 3 to less than 6 months ago
   - ❑ 6 months ago to less than 1 year ago
   - ❑ 1 year ago to less than 3 years ago
   - ❑ 3 years ago to less than 5 years ago
   - ❑ More than 5 years ago

9. When did you have your most recent screening mammogram?
   - ❑ Less than one month ago
   - ❑ 1 to less than 3 months ago
   - ❑ 3 to less than 6 months ago
   - ❑ 6 months ago to less than 1 year ago
   - ❑ 1 year ago to less than 3 years ago
   - ❑ 3 years ago to less than 5 years ago
   - ❑ More than 5 years ago

Experiences with COVID-19

10. Have your friends or family experienced any COVID-19 symptoms such as fever, coughing, upper respiratory distress, or shortness of breath?
    - ❑ No
    - ❑ Yes

11. Have you experienced any COVID-19 symptoms such as fever, coughing, upper respiratory distress, or shortness of breath?
    - ❑ No
    - ❑ Yes

12. Have you been tested for COVID-19?
    - ❑ No (skip to Q14)
    - ❑ Yes (continue)

13. Have you been diagnosed with COVID-19?
    - ❑ No (skip to Q14)
    - ❑ Yes (continue)

14. Have you been hospitalized as a result of COVID-19?
    - ❑ No
    - ❑ Yes

15. How concerned are you that COVID-19 will negatively affect your health?
    - ❑ Not concerned
    - ❑ Somewhat concerned
    - ❑ Very concerned
    - ❑ Extremely concerned

16. Is there a shelter-in-place or stay-at-home order or another local ordinance in place where you live to prevent exposing yourself or someone else to COVID-19?
    - ❑ Yes
    - ❑ No
    - ❑ Don't know

17. What precautions are you taking to prevent exposing yourself or someone else to COVID-19? (Please select all that apply)
    - ❑ Self-quarantine (I do not leave my home or see anyone outside of my household)
    - ❑ I am not seeing anyone outside my house except for essential needs (shopping, etc.)
    - ❑ I work in an essential field, but practicing social distancing outside of work
    - ❑ Seeing my family and friends outside the house but taking general precautions (remaining six feet away, coughing into sleeve, etc.)
    - ❑ I am wearing a mask, gloves, or other protective equipment when I leave my house
    - ❑ Working from home
    - ❑ No changes

Impact of COVID-19 on Breast Cancer Screening, Diagnosis, and Treatment

18. Have you discussed COVID-19 with a health care provider?
    - ❑ No
    - ❑ Yes

19. Which answer best describes the location where you are currently receiving screening, diagnostic evaluation, treatment, or follow-up care?
    - ❑ An academic center (hospital connected with a university)
    - ❑ A regional center (a hospital that serves multiple cities)
    - ❑ A community hospital (a hospital that is primarily used by one town or city)
    - ❑ Private practice

20. Have any health care facilities that you attend for breast cancer screening, diagnostic evaluation, treatment, or follow-up care had to close because of COVID-19?
    - ❑ No
    - ❑ Yes
    - ❑ Don't know

21. Have any of your doctor's visits or treatments been changed, delayed, rescheduled, interrupted, or stopped as a result of COVID-19?
    - ❑ No (skip to Q24)
    - ❑ Yes
    - ❑ Don't know

22. What types of changes, delays, or rescheduling has occurred as result of COVID-19, if any? (select all that apply)
    - ❑ Breast cancer screening, diagnosis, care or treatment has been **delayed** by **less than 2 weeks**
    - ❑ Breast cancer screening, diagnosis, care or treatment has been **delayed** by **more than 2 weeks**
    - ❑ Breast cancer screening, diagnosis, care or treatment has been **delayed** and I **don't know when it will be rescheduled**
    - ❑ Breast cancer screening, diagnosis, care or treatment has been **cancelled** and **I don't expect it to be rescheduled**
    - ❑ My breast cancer treatment plan was changed (for example, I was switched from an infused medication to one that can take in a pill)
    - ❑ Reduced the number of days I go to a healthcare center or hospital for chemotherapy or radiation treatments
    - ❑ Changed the location that I go to for breast cancer screening, diagnosis, care or treatment
    - ❑ A visit that was supposed to be in person has been changed to phone or video
    - ❑ Care than was supposed to be given outside my home has been changed to happen at my home
    - ❑ I received supplies or prescriptions by mail or delivery instead of picking them up in person
    - ❑ I'm not sure
    - ❑ None of the above (SKIP to Q23)

23. Which breast cancer screenings, diagnostic evaluations, or treatments have been delayed, rescheduled, interrupted, or stopped because of COVID-19? (Please mark all that apply)
    - ❑ Mammogram

- ❑ MRI
- ❑ Ultrasound
- ❑ Surgery
- ❑ Radiation
- ❑ Chemotherapy
- ❑ Pills/Hormone therapy
- ❑ Targeted therapy/Infusion but not chemotherapy
- ❑ Clinical trial
- ❑ Nothing has changed
- ❑ Not sure what is going to happen, waiting to talk to my doctor
- ❑ Other, please specify:

24. Have your doctors added additional services to reduce in-person appointments? (Please select all that apply.)
    - ❑ Virtual appointments (video conferences)
    - ❑ Telephone conferences
    - ❑ Email exchanges
    - ❑ Sending pills to you through the mail
    - ❑ No extra services
    - ❑ Other, please specify:

25. Have you or your doctors used any of the following methods to connect with each other during the COVID-19 pandemic? (Please mark all that apply)
    - ❑ Virtual appointments (video conferences)
    - ❑ Telephone conferences
    - ❑ Email
    - ❑ Home visits
    - ❑ Social media
    - ❑ Telephone
    - ❑ Texts
    - ❑ I have not talked to my doctors
    - ❑ Other, please specify:

26. How long does it take you to travel to the center where you are currently receiving screening, diagnostic evaluation, treatment, or follow-up care? (if you fly to your appointments, choose the answer based on how long it would take you by car).
    - ❑ 0-1 hours
    - ❑ 1-2 hours
    - ❑ 3-5 hours
    - ❑ 5+ hours

27. Have any of the following transportation issues related to the COVID-19 pandemic affected your ability to get to health services and appointments?
   - ❑ Public transportation is running on a reduced schedule
   - ❑ Fewer family and friends are available to drive me to appointments
   - ❑ Ride to treatment programs (like ACS Road to Recovery or others) are on a reduced schedule or are no longer available
   - ❑ Some roads are closed, making it difficult to figure out the best way to get there
   - ❑ I'm afraid of being exposed to COVID-19 on public or shared transportation
   - ❑ Rideshare drivers (such as Uber or Lyft) are not as easily available
   - ❑ Travel advisories or flight cancellations due to COVID-19
   - ❑ Other
   - ❑ Transportation challenges have not impacted my ability to get healthcare

28. What aspect of your health care is most affected by COVID-19?

COVID-19 Concerns

29. Rate your anxiety about your cancer progressing or recurring during the COVID-19 pandemic: **(FOR WOMEN WITH BREAST CANCER)**

**Not Anxious**									**Extremely Anxious**
**0**	**1**	**2**	**3**	**4**	**5**	**6**	**7**	**9**	**10**

30. Rate your anxiety during the COVID-19 pandemic: **(FOR WOMEN WHO DON'T HAVE CANCER)**

**Not Anxious**									**Extremely Anxious**
**0**	**1**	**2**	**3**	**4**	**5**	**6**	**7**	**9**	**10**

31. How concerned are you that the COVID-19 pandemic will affect your economic situation?
    - ❑ Not concerned
    - ❑ Somewhat concerned
    - ❑ Very concerned
    - ❑ Extremely concerned

32. In what, if any, ways has the COVID-19 outbreak and its impact changed your financial situation related to your ability to pay for health care? (Please select all that apply.)
    - ❑ I have lost my job, or a member of my family who lives with me has lost his or her job
    - ❑ I, or a family member who lives with me, have had my/their work hours reduced
    - ❑ I have lost or changed health insurance due to changes in my or a family member's job
    - ❑ My retirement, investment, or savings accounts are worth less, and it's affecting my ability to pay for health care expenses
    - ❑ Changes due COVID-19 increased by out-of-pocket costs for care (for example, my doctor suggested I get a 90-day supply of medication, but it was hard to afford the higher cost)
    - ❑ It was difficult to afford the food and household supplies I needed to make sure I could stay home to avoid exposure to COVID-19
    - ❑ Other
    - ❑ No notable impact on my financial situation and ability to pay for care

33. To what extent do you agree or disagree with the following statements? **(FOR WOMEN WITH BREAST CANCER)**

	**Strongly agree**	**Agree**	**Neither agree nor disagree**	**Disagree**	**Strongly disagree**	**N/A**
a. I know where to get accurate information about COVID-19 that is specific to me as a breast cancer patient or survivor						
b. I am worried that the COVID-19 pandemic and the response to it will make it harder for me to get treatment for my cancer						
c. I am worried that the financial impact of COVID-19 will make it hard for me to afford the cancer care I need						
d. As a breast cancer patient or survivor, if I am infected with COVID-19, I am at a higher risk of serious illness and complications						

34. To what extent do you agree or disagree with the following statements? **(FOR WOMEN WHO DON'T HAVE CANCER)**

	Strongly agree	Agree	Neither agree nor disagree	Disagree	Strongly disagree	N/A
a. I know where to get accurate information about COVID-19						
b. I am worried that the COVID-19 pandemic and the response to it will make it harder for me to get health care, such as screening or diagnostic evaluation						
c. I am worried that the financial impact of COVID-19 will make it hard for me to afford the health care I need						

35. What is your biggest concern as a cancer patient during COVID-19? **(FOR WOMEN WITH BREAST CANCER)**
36. What is your biggest concern during COVID-19? **(FOR WOMEN WHO DON'T HAVE CANCER)**

Personal Characteristics

37. Has a first-degree relative (i.e. mother, sister, daughter) ever been diagnosed with breast cancer?
- ❑ No
- ❑ Yes
- ❑ Prefer not to say

38. What is your sex?
- ❑ Male
- ❑ Female
- ❑ Non-Binary
- ❑ Prefer not to say

39. Do you identify as transgender?
- ❑ No
- ❑ Yes
- ❑ Prefer not to say

40. What is your age?
- ❑ 18 to 24 years old
- ❑ 25 to 34 years old
- ❑ 35 to 39 years old
- ❑ 40 to 44 years old
- ❑ 45 to 49 years old
- ❑ 50 to 54 years old
- ❑ 55 to 59 years old
- ❑ 60 to 64 years old
- ❑ 65 to 69 years old
- ❑ 70 to 74 years old
- ❑ 75 years or older
- ❑ Prefer not to say

41. Which of the following best describes your race? (Please select all that apply):
- ❑ White
- ❑ Hispanic or Latino
- ❑ Black or African American
- ❑ Asian
- ❑ Native Hawaiian or other Pacific Islander
- ❑ American Indian or Alaskan Native
- ❑ Other: ___________________________
- ❑ Prefer not to say

42. Are you of Hispanic, Latino, or Spanish origin?
- ❑ No
- ❑ Yes
- ❑ Prefer not to say

43. Are you of Ashkenazi Jewish descent?
- ❑ No
- ❑ Yes
- ❑ Prefer not to say

44. What is the highest degree or level of school you have completed?
- ❑ Less than high school diploma
- ❑ High school degree or similar (e.g. GED)
- ❑ Some college, no degree
- ❑ Associate degree
- ❑ Bachelor's degree
- ❑ Master's degree
- ❑ Professional degree (MD, DDS) or Doctorate (PhD)
- ❑ Prefer not to say

45. Which region of the United States do you live in?
- ❑ New England - Connecticut, Maine, Massachusetts, New Hampshire, Rhode Island, Vermont
- ❑ Middle Atlantic - New Jersey, New York, Pennsylvania
- ❑ East North Central - Illinois, Indiana, Michigan, Ohio, Wisconsin
- ❑ West North Central - Iowa, Kansas, Minnesota, Missouri, Nebraska, North Dakota, South Dakota
- ❑ South Atlantic - Delaware, District of Columbia, Florida, Georgia, Maryland, North Carolina, South Carolina, Virginia, West Virginia
- ❑ East South Central - Alabama, Kentucky, Mississippi, Tennessee
- ❑ West South Central - Arkansas, Louisiana, Oklahoma, Texas
- ❑ Mountain - Arizona, Colorado, Idaho, Montana, Nevada, New Mexico, Utah, Wyoming
- ❑ Pacific - Alaska, California, Hawaii, Oregon, Washington
- ❑ I live outside of the United States
- ❑ Prefer not to say

46. Which best describes where you live?
- ❑ Urban (a town or city)
- ❑ Suburban (residential area within commuting distance of a city)
- ❑ Rural (the countryside located outside towns and cities)
- ❑ Prefer not to say

47. Currently, what type of health insurance coverage, if any, do you have? (Please select all that apply)
- ❑ A plan through an employer or union (Private insurance)
- ❑ A plan that you or someone else buys on your own (Self-insured)
- ❑ Medicare
- ❑ Medicaid
- ❑ Some other government insurance
- ❑ Other
- ❑ Not covered by insurance
- ❑ Don't know
- ❑ Prefer not to say

48. What is your approximate household income?
- ❑ $0-$24,999

- ❑ $25,000-$49,999
- ❑ $50,000-$74,999
- ❑ $75,000-$99,999
- ❑ $100,000-$124,999
- ❑ $125,000-$149,000
- ❑ $150,000-$174,999
- ❑ $175,000-$199,999
- ❑ $200,000 and up
- ❑ Prefer not to say

49. Are you willing to be contacted again in the future to follow-up on your experiences with COVID-19?
    - ❑ No
    - ❑ Yes

Thank you for completing the survey. If you would like to participate in the raffle or if you have indicated that you are willing to be contacted for future studies, please provide your name and email address below.

50. What is your first name? ________________________
51. What is your last name? ________________________
52. What is your email address? _________________________

END OF SURVEY

Thank you for your time!
